# Staging, Neurocognition and Social Functioning in Bipolar Disorder

**DOI:** 10.3389/fpsyt.2018.00709

**Published:** 2018-12-19

**Authors:** Amparo Tatay-Manteiga, Patricia Correa-Ghisays, Omar Cauli, Flavio P. Kapczinski, Rafael Tabarés-Seisdedos, Vicent Balanzá-Martínez

**Affiliations:** ^1^Department of Psychiatry, General University Hospital Consortium of Valencia, Valencia, Spain; ^2^Centro Investigación Biomédica en Red de Salud Mental (CIBERSAM), Madrid, Spain; ^3^Faculty of Psychology, University of Valencia, Valencia, Spain; ^4^Department of Nursing, University of Valencia, Valencia, Spain; ^5^McMaster's Department of Psychiatry and Behavioral Neurosciences, Hamilton, ON, Canada; ^6^Department of Psychiatry, Universidade Federal do Rio Grande do Sul, Porto Alegre, Brazil; ^7^Department of Medicine, University of Valencia, Valencia, Spain; ^8^Catarroja Mental Health Unit, Valencia, Spain

**Keywords:** bipolar disorder, clinical staging, functioning, neurocognition, siblings, first-degree relatives

## Abstract

**Introduction:** Bipolar disorder (BD) is associated with significant neurocognitive and functional impairment, which may progress across stages. The ‘latent stage' of BD remains understudied. This cross-sectional study assessed staging, neurocognition and social functioning among BD patients and their healthy siblings.

**Methods:** Four groups were included: euthymic type I BD patients in the early (*n* = 25) and late (*n* = 23) stages, their healthy siblings (latent stage; *n* = 23) and healthy controls (*n* = 21). All 92 subjects underwent a comprehensive neuropsychological battery of processing speed, verbal learning/memory, visual memory, working memory, verbal fluency, executive cognition, and motor speed. Social functioning was assessed using the FAST scale.

**Results:** Siblings' social functioning was identical to that of controls, and significantly better than both early- (*p* < 0.005) and late- (*p* < 0.001) stage patients. Although all patients were strictly euthymic, those at late stages had a significantly worse social functioning than early-stage patients (*p* < 0.001). Compared to controls, increasingly greater neurocognitive dysfunction was observed across stages of BD (*F* = 1.59; *p* = 0.005). Healthy siblings' performance lied between those of controls and patients, with deficits in tasks of processing speed, executive attention, verbal memory/learning, and visual memory. Both early- and late-stage patients had a more severe and widespread dysfunction than siblings, with no significant differences between them.

**Conclusions:** Genetic vulnerability to BD-I seems to be associated with neurocognitive impairments, whereas social dysfunction would be the result of the clinical phenotype. Staging models of BD should take into account these divergent findings in the latent stage.

## Introduction

Bipolar disorder (BD) is a severe, chronic mood disorder characterized by recurrent episodes of depression and (hypo)mania, interspersed with periods of clinical remission or euthymia. BD is associated with an important global disability as well as increased morbidity and mortality (1, 2).

Several neurocognitive deficits have been described during euthymia, including the broad domains of attention/processing speed, verbal learning and memory, and executive functions, such as cognitive flexibility, working memory and verbal fluency (3, 4). Problems in social functioning also persist into euthymia (5, 6) and seem to worsen with more relapses (7). More importantly, persistent neurocognitive deficits have been associated with functional outcomes during euthymia (8, 9).

Accordingly, several clinical staging models have been put forward to classify BD patients into different stages of the disease, taking into account clinical, neurocognitive and functional variables (10, 11). One such model relies on disability during the interepisodic period (12). As predicted by this model, patients in the early stages of the disease would have less neurocognitive and functional deterioration than those in the late stages of BD. Interestingly, this model also encompass a “latent stage,” which includes healthy first-degree relatives of BD patients, who are predicted to show no deterioration in their neurocognitive and social functioning.

On the other hand, the concurrent study of first-degree relatives of patients may allow investigate and identify endophenotypes for BD. Endophenotypes or intermediate phenotypes are features associated with the etiophisiopathology of an illness (13). Suitable endophenotypes should be associated with the disease within a population, be state-independent, be heritable, and co-segregate with the disease within families and found in healthy relatives in a greater proportion than in the general population (14). Therefore, the identification of clinical, neurobiological and functional changes in healthy family members would potentially help identify valid endophenotypes of BD and refine clinical staging, with the ultimate goal of early diagnosis and intervention. There is rising interest in searching neurocognitive endophenotypes associated with BD, since cognition is a major predictor of patients' functional outcomes (9).

Growing research has identified similar, yet milder, neurocognitive dysfunction among healthy relatives of BD patients (3). However, most studies have examined mixed groups of relatives, including parents, siblings, and offspring of BD patients (15). The few studies that have focused specifically on healthy siblings have also found neurocognitive deficits, mostly in verbal memory, visual memory and executive functioning (16–20).

Clinical staging of BD is a relatively new area of research, and staging models need empirical validation (21). This study aimed to explore Kapczinski's staging model based on neurocognitive and functional impairment among BD patients and their healthy siblings, compared to healthy controls.

## Materials and Methods

### Study Sample

An observational, cross-sectional study was carried out comparing neurocognitive and functional performance in four different groups: euthymic BD patients in early stages of the disease; euthymic BD patients in late stages of the disease; healthy subjects with an increased genetic risk for developing BD, in this case, siblings of patients diagnosed with BD (“latent stage”); and healthy subjects without personal nor family history of BD as a control group (22). The sample was recruited at the Doctor Peset University Hospital health department in Valencia, Spain. The study was approved by the hospital Ethics Committee. Written informed consent of the participants was obtained after procedures had been fully explained.

Inclusion criteria were: adults under 60 years old; diagnosed with DSM-IV-TR BD type I; outpatient or living in a residence; clinically euthymic confirmed with psychometric criteria (Hamilton Rating Scale for Depression, HRSD-17 <8; and Young Mania Rating Scale, YMRS <7) for a period of at least 2 months; receiving a stable regimen of medication for at least 4 weeks; and able to understand the study procedures.

Exclusion criteria were: clinical conditions impeding study procedures; current hospitalization; documented cognitive impairment (intellectual disability or dementia); physical, visual or hearing disability that would prevent from understanding the protocol; and inability to read or understand Spanish. Inclusion criteria for relatives and healthy controls were: adults under 60 years old, with no diagnosis of psychiatric disorders on Axis I confirmed by the SCID-I interview, able to understand the procedures of the study and to provide written informed consent. In addition to exclusion criteria applied to patients, relatives had to have a brother or sister diagnosed with BD type I, whereas healthy controls should have no family history of severe mental illness, including schizophrenia or other psychotic disorders, BD and major depressive disorder in first- and second-degree relatives.

### Assessments

Each subject underwent a complete clinical, neuropsychological, and functional assessment. Pre-morbid intelligence quotient (PIQ) was estimated with the WAIS III Vocabulary subtest. Neurocognition was evaluated with a comprehensive neuropsychological battery including the following tests: WAIS III Digit Symbol, COWA test (including the FAS and the Animal Naming test),Wisconsin Card Sorting Test (WCST), Trail Making Test (TMT) parts A and B, Stroop Color and Word Test, WAIS III Digit Span, California Verbal Learning Test (CVLT), Rey-Osterrieth Complex Figure test (ROCFT), and Finger Tapping Test (FTT) [for details, see (9, 23, 24)]. The battery taps on tasks of processing speed, abstract reasoning, cognitive flexibility, verbal fluency, selective attention, working memory, verbal learning/memory, visual memory, and motor speed, which are the most relevant domains in neurocognitive assessment of BD patients and their families (4, 15). As a result, 23 neurocognitive variables were obtained.

Social functioning was assessed with the Functional Assessment Short Test [FAST, (25)]. The FAST is an easy to use scale to evaluate difficulties that patients have in their daily lives. It has 24 items grouped in 6 domains or specific areas of functioning: autonomy (ability to do things alone and make one's own decisions), work performance (ability to maintain a paid job, efficiency in carrying out tasks at work), cognitive functioning (ability to concentrate, performing simple mental calculations, solving problems, learning and remembering new information), finance (ability to manage finances and spend in a balanced way), relationships (relationships with friends, family, participation in social activities, sexual relations) and leisure (ability to perform sports, exercise or enjoy hobbies). The scores for each item range from 0 to 3. The overall score is obtained by adding the scores of each item. The higher the score, the greater the difficulty in patient's functioning. The median value of the FAST total score has been used to classify BD outpatients into two broad groups based on Kapczinski's clinical staging model: early- and late-stages (26).

### Statistical Analysis

Socio-demographic, clinical, functional, and neurocognitive variables were analyzed using descriptive statistics, with a confidence interval of 95% to two tails in both cases. Parametric and non-parametric tests were used for the analysis of continuous variables depending on the restrictions of applicability (normality) and the nature of the variable. The association between categorical variables was analyzed with the Pearson chi-square test (χ^2^) or Fisher's exact test, as appropriate.

Continuous variables were compared using the *t* Student test or the analysis of variance (ANOVA). For comparisons of parametric variables between more than two groups, if the main effect was significant, pair-wise comparisons were performed by *post-hoc* tests (Scheffé). To analyze the differences between clinical groups (e.g., early and late stages) *t* test for independent samples was used. To compare non-parametric variables between independent samples, the Mann-Whitney test was used. Analysis of covariance (MANCOVA) was used to control the influence of confounding variables. The last version of SPSS software program (SPSS Inc., Chicago, USA) was used and statistical significance for all tests was set at *p* < 0.05.

## Results

### Study Sample

A total of 107 subjects (53 patients, 26 siblings, and 28 controls) were initially recruited. Of them, 15 were excluded for several reasons: two subjects did not complete the neurocognitive assessment; three subjects met exclusion criteria: two had a family history of mental illness (BD and schizophrenia, respectively) and one had a personal history of mental illness (major depression); two subjects were excluded for providing insufficient blood sample; and eight subjects for laboratory reasons.

The final sample consisted of 92 individuals divided into four groups: 25 early-stage patients, 23 late-stage patients, 23 siblings of BD-I patients and 21 healthy controls. Based on previous studies (26), the criterion used to classify patients into early and late stages was the median of the FAST scale, in this case 32 points.

### Sample Description

A complete sample description can be found in a previous article (22). Demographic characteristics of the four groups are described in Table 1. There were no significant differences between groups in most socio-demographic variables, including sex, age, marital status, and living status. However, the groups significantly differed in their occupational status, as expected. Moreover, there were significant between-groups differences in years of education and estimated PIQ. As these two variables were correlated (*r* = 0.49; *p* < 0.001), education was used for the remaining analyses. In addition, the four groups significantly differed in the presence of residual mood symptoms both depressive and manic (Table 1).

**Table 1:** Sample description.

**Variables^**a**^**	**Controls** **(*n* = 21)**	**Healthy siblings** **(*n* = 23)**	**Early-stage BD** **(*n* = 25)**	**Late-stage BD** **(*n* = 23)**	**F^**b, c, d**^**	***P***	***post-hoc***
**Sex ratio**							
(male: female)	7:14	7:16	12:13	11:12	2.49^**b**^	0.49	–
Age (y)	36.7 ± 10,9	41.5 ± 11,8	43.4 ± 10.3	45.1± 9.8	2,49^**c**^	0,07	–
Education (y)	14.3 ± 3.1	12.6 ± 2.9	11.8 ± 3	11.8 ± 2.9	3.48^**c**^	**0.019**	C>E,L
Premorbid IQ	123.1 ± 10.3	111.5 ± 12.6	107.4 ± 11.7	109.6 ± 10.8	8.27^**c**^	**<0.0001**	C>S,E,L
**Marital status**, ***n*** **(%)**							
Single	11 (52.4%)	9 (39.1%)	11 (44%)	8 (34.8%)	12.91^**b**^	0.11	–
Married	9 (42.9%)	12 (52.2%)	7 (28%)	12 (52.2%)			
Widow	1 (4.8%)	0 (0%)	0 (0%)	0 (0%)			
Separated	0 (0%)	2 (8.7%)	7 (28%)	3 (13%)			
**Living status**, ***n*** **(%)**							
Alone	3 (14.3%)	2 (8.7%)	5 (20%)	2 (8.7%)	8.78^**b**^	0.73	–
Parents	6 (28.6%)	5 (21.7%)	4 (16%)	2 (8.7%)			
Partner	5 (23.8%)	4 (17.4%)	7 (28%)	6 (26.1%)			
Own family	7 (33.3%)	12 (52.2%)	8 (32%)	12 (52.2%)			
Other	0 (0%)	0 (0%)	1 (4%)	1 (4.3%)			
**Completed education**, ***n*** **(%)**							
Primary	3 (14.3%)	6 (26.1%)	10 (40%)	9 (39.1%)	8.68^**b**^	0.19	–
Secondary	7 (33.3%)	10 (43.5%)	9 (36%)	10 (43.5%)			
University	11 (52.4%)	7 (30.4%)	6 (24%)	4 (17.4%)			
**Occupationstatus**, ***n*** **(%)**							
Student	5 (23.8%)	2 (8.7%)	0 (0%)	0 (0%)	55.69^**b**^	**<0.0001**	
Housework	1 (4.8%)	1 (4.3%)	0 (0%)	0 (0%)			
Employed	11 (52.4%)	15 (65.2%)	5 (20%)	2 (8.7%)			
Sick leave	0 (0%)	1 (4.3%)	1 (4%)	1 (4.3%)			
Retired	0 (0%)	0 (0%)	13 (52%)	14 (60.9%)			
Unemployed	4 (19%)	4 (17.4%)	6 (24%)	6 (26.1%)			
HRSD total	1.6 ± 1.4	0.9 ± 1.3	2.0 ± 1.9	3.3 ± 1.8	8.85 c	**<0.0001**	L>C,S,E
YMRS total	0.7 ± 0.9	0.3 ± 0.6	1.1 ± 1.3	1.5 ± 1.6	4.24 d	**0.008**	L>S

a *Expressed as mean ± standard deviation*.

b *Fishertest*.

c *ANCOVA*.

d *ANOVA. C, control; E, early stage; HRSD, Hamilton Rating Scale for Depression; L, late stage; S, sibling; YMRS, Young Mania Rating Scale. Bold values are to remark statistically significant results*.

Early-stage patients were comparable to late-stage patients since no significant differences in any of the pharmacological or clinical variables analyzed were found (Table 2).

**Table 2:** Clinical and pharmacological variables of early- and late-stage BD groups.

**Variables^**a**^**	**Early stage (*n* = 25)**	**Late stage (*n* = 23)**	***t*-test**	***p***
Age of onset	23.0 ± 7.0	26.9 ± 7.9	0.1	0.07
Illness duration	20.1 ± 10.9	18.0 ± 9.7	0.09	0.5
Number of episodes (total)	24.8 ± 49.9	11.0 ± 8.9	5.89	0.18
Number of admissions (total)	4.4 ± 7.8	3.8 ± 6.5	0.1	0.8
Time since last episode (months)	26.8 ± 36.1	13.1 ± 19.0	6.5	0.11
Time since last admission (months)	42.6 ± 49.9	28.1 ± 45.2	0.57	0.3
Tobacco smoking, Yes, *n* (%)	12 (48%)	15 (65.2%)	1.44	0.26
Rapid cycling, Yes, *n* (%)	2 (8%)	3 (13%)	0.33	0.66
Seasonal pattern, Yes, *n* (%)	4 (16%)	4 (17.4%)	0.17	1
Psychotic symptoms, Yes, *n* (%)	16 (64%)	17 (73.9%)	0.55	0.54
Suicide ideation, Yes, *n* (%)	18 (72%)	16 (69.6%)	0.03	1
Suicide attempt, Yes, *n* (%)	6 (24%)	6 (26.1%)	0.03	1
Lithium, Yes, *n* (%)	18 (72%)	13 (56.5%)	1.25	0.37
Valproate, Yes, *n* (%)	4 (16%)	8 (34.8%)	2.25	0.19
Lithium, mean daily dose ^b^	696.0 ± 538.9	530.4 ± 499.5	1.17	0.28
Valproate, mean daily dose ^b^	220.0 ± 541.6	404.3 ± 619.0	2.43	0.28
Number of medications	2.9 ± 1.5	3.0 ± 1.6	0.39	0.77

a Expressed as mean ± standard deviation, except when indicated

b *Dose expressed in mg/day*.

### Comparison of Neurocognitive Functioning

First, neurocognitive performance of the four groups was compared by analysis of covariance (MANCOVA), controlling for covariates that may influence neurocognition, such as age, education and residual depressive and manic symptoms. Age (Pillai Trace: *F* = 3.51; *p* < 0.0001), education (*F* = 1.82; *p* = 0.03) and group (*F* = 1.51; *p* = 0.013) exerted a major effect, while depressive symptoms (*F* = 1.08, *p* = 0.39) and manic symptoms (*F* = 0.46, *p* = 0.98) had no influence on neurocognitive performance. Therefore, age and education were used as covariates in the remaining analyses of this section. After controlling the influence of these covariates, there were statistically differences between groups in overall neurocognitive functioning (MANCOVA: *F* = 1.59, *p* = 0.005) (Table 3).

**Table 3:** Neurocognitive functioning of the four groups.

**Variables^**a**^**	**Controls (*n* = 21)**	**Healthy siblings (*n* = 23)**	**Early stage BD (*n* = 25)**	**Late stage BD (*n* = 23)**	**F^**b**^**	***P***
Digit Symbol	87.9 ± 20.9	77.7 ± 16.2	59.1 ± 20.4^*^^§^	55.8 ± 19.7^*^^§^	9.46	**<0.0001**
Animal Naming Test	25.9 ± 6.2	22.8 ± 5.0	20.6 ± 6.3	21.0 ± 5.8	1.33	0.27
FAS	47.9 ± 9.1	43.3 ± 9.4	38.3 ± 16.7	39.6 ± 10.6	0.7	0.56
**WCST**						
Total errors	14.2 ± 7.1	21.8 ± 15.2	35.7 ± 23.4^*^^§^	38.1 ± 21.6^*^^§^	4.83	**0.004**
Perseverations	8.3 ± 4.9	13.0 ± 10.4	26.9 ± 22.5^*^^§^	26.3 ± 21.9^*^^§^	3.8	**0.013**
Perseverative Errors	7.8 ± 4.1	12.0 ± 9.1	23.1 ± 18.2^*^^§^	22.3 ± 17.0^*^^§^	3.79	**0.013**
Non Perseverative Errors	6.5 ± 3.2	9.9 ± 6.7	12.5 ± 7.6	15.8 ± 10.3^*^^§^	3.18	**0.028**
Categories	5.9 ± 0.4	5.7 ± 0.8	4.7 ± 1.6^§^	4.4 ± 1.9^*^^§^	4.04	**0.01**
**TMT**						
Part A (TMT-A)	21.8 ± 4.0	33.4 ± 10.1*	36.8 ± 16.7*	42.9 ± 17.2^*^^§^	5.17	**0.002**
Part B (TMT-B)	49.9 ± 14.2	66.3 ± 24.1*	84.1 ± 36.4^*^^§^	101.9 ± 45.2^*^^§^	6.0	**0.001**
**Stroop Test**						
Word	113.1 ± 14.5	109.6 ± 11.4	101.8 ± 19.7	97.0 ± 21.8^§^	1.11	0.35
Colour	78.9 ± 10.8	71.5 ± 8.5*	64.6 ± 12.0^*^^§^	64.0 ± 16.5*	3.07	**0.032**
Word-Colour	49.7 ± 9.7	44.0 ± 8.0	37.2 ± 11.1^*^^§^	39.7 ± 13.7	2.25	0.09
Digits forward	8.8 ± 1.8	8.9 ± 1.7	7.9 ± 2.0	8.1 ± 1.7	0.83	0.48
Digits backward	6.9 ± 1.6	6.9 ± 1.7	5.2 ± 1.8^*^^§^	5.0 ± 1.7^*^^§^	5.57	**0.002**
**CVLT**						
Total learning	60.6 ± 7.3	54.0 ± 6.5*	46.7 ± 10.7^*^^§^	43.3 ± 10.7^*^^§^	11.47	**<0.0001**
Immediate verbal recall	27.3 ± 2.7	24.4 ± 4.4*	19.5 ± 6.1^*^^§^	17.8 ± 5.5^*^^§^	12.51	**<0.0001**
Delayed verbal recall	28.6 ± 2.8	24.7 ± 4.6*	19.5 ± 6.5^*^^§^	17.9 ± 6.0^*^^§^	14.47	**<0.0001**
Total recognition	43.3 ± 0.8	41.3 ± 2.1*	40.0 ± 2.9*	38.8 ± 3.0^*^^§^	9.62	**<0.0001**
**ROCFT**						
Immediate visual recall	24.9 ± 4.8	19.6 ± 6.5*	16.6 ± 8.0*	15.3 ± 8.0^*^^§^	4.87	**0.004**
Delayed visual recall	24.7 ± 5.4	19.7 ± 6.3*	15.9 ± 8.5*	15.2 ± 7.8^*^^§^	4.74	**0.004**
**FTT**						
Unimanual	95.0 ± 16.0	97.4 ± 20.9	82.8 ± 15.1^*^^§^	77.7 ± 22.8^§^	4.31	**0.007**
Bimanual	88.1 ± 16.6	91.8 ± 20.7	76.5 ± 13.2^§^	73.8 ± 23.4^§^	3.6	**0.017**

* p < 0.05 vs. controls (for the remaining three groups)

§ p < 0.05 vs. healthy siblings (for both clinical groups)

a *Raw performances expressed as mean ± standard deviation*.

b *Main effect of factor “group” in MANCOVA with education and age as covariates. Between-group significant differences appear in bold. CVLT, California Verbal Learning Test; FTT, Finger Tapping Test; ROCFT, Rey-Osterrieth Complex Figure Test; TMT, Trail Making Test; WCST, Wisconsin Card Sorting Test*.

Secondly, neurocognitive performance of the four groups were compared pair-wise, as follows: siblings vs. controls, early-stage patients vs. controls, late-stage patients vs. controls, early-stage patients vs. siblings, late-stage patients vs. siblings and both groups of patients. When significant differences existed between the contrasted groups in age, sex, and education, they were introduced as covariates in these pair-wise analyses, as appropriate.

#### Siblings vs. Controls

Both groups were compared by analysis of covariance (ANCOVAs) with education as a covariate (*Z* = −1.99; *p* = 0.047). Compared to controls, siblings had a significantly worse performance (*p* < 0.05) and therefore a deficit, in 9 of the 23 variables analyzed: TMT-A, TMT-B, Stroop color, CVLT (total learning, immediate and delayed verbal recall, recognition), and immediate and delayed visual recall.

#### Early-Stage Patients vs. Controls

Neurocognitive performance of both groups was compared by ANCOVA with age (*Z* = −2.03; *p* = 0.042) and education (*Z* = −2.58; *p* = 0.01) as covariates. Compared to healthy controls, early-stage patients showed a deficit in 16 variables: digit symbol, WCST (total errors, perseverations, perseverative errors), TMT-A, TMT-B, Stroop color, Stroop word-color, digits backward, CVLT (total learning, immediate and delayed verbal recall, recognition), immediate and delayed visual recall, and FTT unimanual.

#### Late-Stage Patients vs. Controls

Neurocognitive performance of both groups was compared by ANCOVA with age (*Z* = −2.55; *p* = 0.011) and education (*Z* = −2.57; *p* = 0.01) as covariates. Compared with healthy controls, late-stage patients had a deficit in 16 variables: digit symbol, WCST (total errors, perseverations, perseverative errors, non-perseverative errors, categories), TMT-A, TMT-B, Stroop color, digit backward, CVLT (total learning, immediate and delayed verbal recall, recognition), and immediate and delayed visual recall.

#### Early-Stage Patients vs. Siblings

Since both groups were comparable in age, sex, and education, the Mann-Whitney test was used, and results were confirmed by *t*-test. Compared to siblings, early-stage patients had significantly worse performance (*p* < 0.05) in 14 variables: digit symbol, WCST (total errors, perseverations, perseverative errors, categories), TMT-B, Stroop color, Stroop word-color, digits backward, CVLT (total learning, immediate and delayed verbal recall), and FTT unimanual and bimanual.

#### Late-Stage Patients vs. Siblings

As in the previous case, performance was compared using the Mann-Whitney test, and results were confirmed by *t*-test. Compared to siblings, late-stage patients had significantly worse performance (*p* < 0.05) in 18 variables: digit symbol, WCST (total errors, perseverations, perseverative errors, non-perseverative errors, categories), TMT-A, TMT-B, Stroop word, digit backward, CVLT (total learning, immediate and delayed recall, recognition), immediate and delayed visual recall, as well as FTT unimanual and bimanual.

#### Early-Stage vs. Late-Stage Patients

We used Mann-Whitney test to compare both groups with no covariates because the two groups were comparable in age, sex, and education. The results were confirmed by *t*-test. No significant differences between both groups of patients were detected in any of the neurocognitive variables examined.

### Analysis of Social Functioning

After controlling for covariates (age, education, and residual affective symptoms), significant between-groups differences (*p* < 0.0001) were found in terms of social functioning measured with the FAST scale. Late-stage patients had a worse performance than early-stage patients, as expected. Moreover, both clinical groups had a worse performance than healthy siblings and controls. Finally, siblings' social functioning was comparable to that of controls (Table 4).

**Table 4:** Comparison of functionality between groups.

**Variables^**a**^**	**Controls (*n* = 21)**	**Healthy siblings (*n* = 23)**	**Early stage (*n* = 25)**	**Late stage (*n* = 23)**	**F^**b**^**	***p***	***post-hoc***
FAST Total	13 ± 9.3	13 ± 10.4	22 ± 6.8	42.2 ± 7.9	32.38	**<0.0001**	L>C,S,E E>C,S
FAST autonomy	1.2 ± 1.4	1.3 ± 1.9	2.4 ± 2.9	4.8 ± 2.5	4.48	**0.006**	L>C,S,E
FAST work	5 ± 5.2	4.7 ± 6.4	8.6 ± 5.4	13.8 ± 3	8.81	**<0.0001**	L>C,S,E
FAST cognition	1.6 ± 1.5	2.6 ± 2.6	4.4 ± 2.8	8.8 ± 3.3	21.08	**<0.0001**	L>C,S,E E>C
FAST finance	0.6 ± 1.5	0.6 ± 1.6	0.9 ± 1.6	3 ± 2.5	5.26	**0.002**	L>C,S,E
FAST relationships	2 ± 1.9	2.2 ± 2.1	3.5 ± 2.9	8.4 ± 3.7	14.82	**<0.0001**	L>C,S,E
FAST leisure	2.6 ± 2.3	1.5 ± 1.9	2.3 ± 2.2	3.3 ± 2.4	1.25	0.3

a *Expressed as mean ± standard deviation*.

b *Main effect of the factor “group” in ANCOVA with years of education and subsyndromic depressive and manic symptoms as covariates. C, control; E, early stage; FAST, Functional Assessment Short Test; L, late stage; S, sibling. Bold values are to remark statistically significant results*.

Differences between groups were significant both in FAST total score and most domains (autonomy, work performance, cognitive functioning, finance, relationships). *Post-hoc*, pair-wise analyses revealed that late-stage patients consistently had a worse global functioning than the other three groups, which in turn did not differ from each other overall. However, early-stage patients showed worse scores on the cognitive domain of the FAST than the controls but similar to those of their siblings (Table 4).

## Discussion

This cross-sectional study showed that the clinical stages of BD seem to be associated with increasing levels of neurocognitive and social dysfunction. The “latent stage” of BD would be present with milder and more restricted neurocognitive deficits, although with spared social functioning. Pervasive neurocognitive dysfunction during euthymia would not worsen with illness progression since early- and late-stage BD-I patients had similar neurocognitive deficits.

### Neurocognitive Functioning

Compared to their healthy siblings and controls, both early and late-stage euthymic BD-I patients had a widespread neurocognitive dysfunction, namely in the domains of processing speed, visual memory, verbal learning/memory, working memory, and executive cognition, which concurs with the literature (3, 4, 27).

Overall, late-stage patients had a similar neurocognitive dysfunction than early-stage patients. According to staging models and the neuroprogression hypothesis of BD, the former group would have been expected to perform significantly worse than the latter (11, 28, 29). However, very few cross-sectional examinations of neurocognition according to clinical staging have been published (26, 30). In a first report, late-stage (stages III and IV) patients showed deficits in verbal learning/memory, working memory and executive attention, whereas early-stage (stages I and II) patients performed similarly to healthy controls (26). A subsequent comparison of the extreme stages revealed that patients on stage I performed significantly better than those on stage IV on verbal learning and memory (30). Overall, the results of these similar studies are at odds with the present findings. Several methodological differences may explain such inconsistent findings. First, previous studies classified BD patients into stages based on clinical judgment (26) or patients' self-report (30), but not according to FAST scores. Second, those studies were conducted at a tertiary center, where patients usually show the more chronic and severe forms of BD (31). Third, the neuropsychological batteries used in previous studies included four and only one test, respectively, therefore our study has used the most comprehensive battery so far. Fourth, in the present study, both clinical groups were matched on demographic, clinical, and treatment terms. Lastly, patients were truly euthymic and had low level of subsyndromic mood symptoms.

The clinical progression of severe mood disorders has been investigated only superficially (32). Moreover, whether neurocognitive dysfunction worsens in parallel with illness progression or not is a hot area of debate (33). There is evidence in favor (11, 34, 35) and against a progressive neurocognitive decline in BD (36–38). According to recent meta-analytic evidence, cognitive impairments in first-episode BD are similar to those shown by multi-episode patients (39). Overall, the neurocognitive findings of this cross-sectional study support the growing understanding that clinical progression is not a general rule in BD, but instead would apply only to a subset of patients (33). A combination of neurodevelopmental and neuroprogressive mechanisms might explain the marked neurocognitive heterogeneity found among BD subjects (40, 41). Indeed, staging and heterogeneity at multiple levels (genetics, clinical, neurocognition) may represent complementary approaches to classify patients (21). Nevertheless, long-term follow-up studies combining neuropsychological, staging and machine learning strategies will shed more light on this controversy (42).

Healthy siblings also showed a relatively wide neurocognitive dysfunction, which encompassed processing speed, executive attention, verbal learning/memory, and visual memory. Overall, the present results concurs with those of the few neurocognitive studies with homogenous samples of healthy siblings of BD patients (16–20, 43). For instance, healthy siblings have been found to show deficits in verbal learning/memory, visuospatial memory, planning and executive attention (18), visual and verbal memory (17, 19) and measures of executive cognition (16, 20).

Most neurocognitive studies of unaffected relatives have focused on offspring or mixed samples of first-degree relatives including parents, siblings and offspring (15). Compared to offspring, healthy siblings and parents of BD patients have a lower risk to develop BD and could be even considered to be resilient to BD, especially those who have gone past the peak age of illness onset. Recent evidence suggests that specific neural mechanisms, such as enhanced integration of the default mode network, may support resilience or delay illness onset among healthy siblings (44). Therefore, the finding of a relatively wide neurocognitive dysfunction in healthy siblings is remarkable. In our study, siblings were healthy subjects as they did not have a personal history of psychiatric disorders on Axis I confirmed by the SCID-I interview and the mean age was later than the usual age onset in BD patients. However, growing research has identified neurocognitive dysfunction among healthy relatives of BD patients (3). Specifically, neurocognitive deficits have been found in healthy siblings, mostly in verbal memory, visual memory and executive functioning (16–20). Obviously, depending on their age, some adult relatives (offspring > siblings > parents) may still face a risk, but most carry unexpressed BD susceptibility genes. This is the reason why siblings are considered as resilient subjects in BD disorder. Overall, they performed intermediate to their affected family members and healthy controls. This converges with the previous literature clearly showing that BD patients have wider and greater neurocognitive impairment than unaffected relatives, and therefore it may represent an endophenotypic marker of BD (3, 15, 24).

On the other hand, the presence of neurocognitive dysfunctions in healthy siblings is at odds with the staging model under evaluation, which predicts spared neurocognition in at-risk subjects (12). However, the “latent stage” clearly remains as an understudied area of research. Recent studies and reviews have revealed several deficits during the premorbid and prodromal phases of BD (39, 45). Since this is the first study comparing the neurocognitive status associated with each stage of the illness, including the latent stage, more research is needed to further explore staging models and advance our understanding of the progressive nature, or lack thereof, of neurocognitive dysfunction in BD.

### Social Functioning

Both clinical groups had a worse social functioning than siblings and controls. Results were consistent at a global level of functioning and also at the majority of functional domains measured by the FAST scale. As predicted by definition, the late-stage was associated with a worse functioning than the early-stage of BD. Of note, siblings' functioning was similar to that of controls. These results converge with those of previous examinations of Kapczinski et al.'s staging model, which also found a progressive worsening of functioning along the stages of BD (7, 25, 46).

Taken together, our results support the prediction of the model regarding the absence of functional impairment in the latent stage, but are at odds concerning neurocognitive dysfunction. In other words, the social dysfunction associated with BD appears to be the result of the clinical phenotype whereas neurocognitive deficits seem to be associated with genetic vulnerability to BD. Future studies comparing patients and unaffected relatives across the stages of BD should include functional assessments with the FAST or similar scales. If confirmed, staging models of BD should be reformulated taking into account the divergence between neurocognitive and social functioning in the latent stage. In this regard, the staging concept needs to be reconciled with neurocognitive dysfunction as an endophenotype of BD (21).

### Limitations and Strengths

The results of the present study must be seen in the context of several *limitations*. Firstly, different criteria have been used to classify patients into the broadly defined early and late stages of BD, and currently no consensus exists about the best choice. Several clinical variables, such as illness duration, comorbidities and functioning, were used in the most recent studies (30, 46, 47), whereas Rosa and colleagues (16) used a cut-off of the FAST in addition. In the pioneer explorations of the model (48, 49), the samples were split according to illness duration into early stage (<3 years since the first manic episode) and late stage (10 years after BD diagnosis). Future research should establish the gold standard to better define early and late stages of BD. Secondly, the influence of other variables, such as medication and comorbidities, on neurocognition was not controlled (50, 51). Other limitations are common in this field, including the cross-sectional design and the small sample size. Despite finding significant differences in most of the between-group analyses, the lack of sample size calculation may have rendered our study underpowered to find additional differences, e.g., false negative results. Nevertheless, the *strengths* and innovations of this study are (i) the inclusion of unaffected relatives and therefore a comprehensive examination of all the stages of the model; (ii) the strict definition of euthymia; (iii) the simultaneous evaluation of the neurocognitive and social functioning with adequate instruments; and (iv) the recruitment of patients at a non-tertiary center, who might be closer to “real world” patients.

## Conclusions

The clinical stages of BD-I seem to be associated with increasing levels of neurocognitive and social deterioration. However, a progressive neurocognitive trajectory does not seem to be a universal phenomenon in BD. Moreover, genetic vulnerability to BD-I would be associated with specific neurocognitive impairments, whereas social dysfunction appears to be the result of the clinical phenotype. This divergent pattern of neurocognitive and social functioning in the latent stage of BD merits further research. More studies, ideally longitudinal, with larger samples and using big data analyses are needed to further explore the empirical validity of staging models of BD.

## Ethics Statement

This study was approved by the Ethics Committee of Dr Peset University Hospital in Valencia, Spain, and was carried out in accordance with the basic principles of the Helsinki Declaration. All subjects gave written informed consent in accordance with the Declaration of Helsinki.

## Author Contributions

AT-M is the principal investigator in the research and has worked in all phases of the study and manuscript preparation. PC-G has participated in data collection and edition of the manuscript. OC has participated in the design and supervision of the study and edition of the manuscript. FK and RT-S has participated as a consultant in the study design and edition of the manuscript. VB-M has participated in the design of the study, supervision of the statistical analysis, and writing and edition of the manuscript. All authors approved the final version of the manuscript.

### Conflict of Interest Statement

The authors declare that the research was conducted in the absence of any commercial or financial relationships that could be construed as a potential conflict of interest.

## References

[1] Soreca I Frank E Kupfer DJ. The phenomenology of bipolar disorder: what drives the high rate of medical burden and determines long-term prognosis? Depress Anxiety (2009) 26:73–82. 10.1002/da.20521 18828143 PMC3308337

[2] Vieta E Popovic D Rosa AR Solé B Grande I Frey BN. The clinical implications of cognitive impairment and allostatic load in bipolar disorder. Eur Psychiatry (2013) 28:21–9. 10.1016/j.eurpsy.2011.11.007 22534552

[3] Bora E Yucel M Pantelis C. Cognitive endophenotypes of bipolar disorder: a meta-analysis of neuropsychological deficits in euthymic patients and their first-degree relatives. J Affect Disord. (2009) 113:1–20. 10.1016/j.jad.2008.06.009 18684514

[4] Bourne C Aydemir Ö Balanzá-Martínez V Bora E Brissos S Cavanagh JT. Neuropsychological testing of cognitive impairment in euthymic bipolar disorder: an individual patient data meta-analysis. Acta Psychiatr Scand. (2013) 128:149–62. 10.1111/acps.12133 23617548

[5] Grande I Magalhães PV Chendo I Stertz L Panizutti B Colpo GD. Staging bipolar disorder: clinical, biochemical, and functional correlates. Acta Psychiatr Scand. (2014) 129:437–44. 10.1111/acps.12268 24628576

[6] Rosa AR Bonnín CM Vázquez GH Reinares M Solé B Tabarés-Seisdedos R. Functional impairment in bipolar II disorder: is it as disabling as bipolar I? J Affect Disord. (2010) 127:71–6. 10.1016/j.jad.2010.05.014 20538343

[7] Rosa AR González-Ortega I González-Pinto A Echeburúa E Comes M Martínez-Aran A. One-year psychosocial functioning in patients in the early vs late stage of bipolar disorder. Acta Psychiatr Scand. (2012) 125:335–41. 10.1111/j.1600-0447.2011.01830.x 22283440

[8] Martino DJ Marengo E Igoa A Scápola M Ais ED Perinot L. Neurocognitive and symptomatic predictors of functional outcome in bipolar disorders: a prospective 1 year follow-up study. J Affect Disord. (2009) 116:37–42. 10.1016/j.jad.2008.10.023 19033081

[9] Tabarés-Seisdedos R Balanzá-Martínez V Sánchez-Moreno J Martínez-Arán A Salazar-Fraile J Selva-Vera G. Neurocognitive and clinical predictors of functional outcome in patients with schizophrenia and bipolar I disorder at one-year follow-up. J Affect Disord. (2008) 109:286–99. 10.1016/j.jad.2007.12.234 18289698

[10] Berk M Hallam KT McGorry PD. The potential utility of a staging model as a course specifier: a bipolar disorder perspective. J Affect Disord. (2007) 100:279–81. 10.1016/j.jad.2007.03.007 17433450

[11] Kapczinski F Magalhães PV Balanzá-Martinez V Dias VV Frangou S Gama CS. Staging systems in bipolar disorder: an international society for bipolar disorders task force report. Acta Psychiatry Scand. (2014) 130:354–63. 10.1111/acps.12305 24961757

[12] Kapczinski F Dias VV Kauer-Sant'Anna M Frey BN Grassi-Oliveira R Colom F. Clinical implications of a staging model for bipolar disorders. Expert Rev Neurother. (2009) 9:957–66. 10.1586/ern.09.31 19589046

[13] Hasler G Drevets WC Gould TD Gottesman II Manji HK Toward constructing an endophenotype strategy for bipolar disorders. Biol Psychiatry (2006) 15:93–105. 10.1016/j.biopsych.2005.11.006 16406007

[14] Gottesman II Gould TD. The endophenotype concept in psychiatry: etymology and strategic intentions. Am J Psychiatry (2003) 160:636–45. 10.1176/appi.ajp.160.4.636 12668349

[15] Balanzá-Martínez V Rubio C Selva-Vera G Martinez-Aran A Sánchez-Moreno J Salazar-Fraile J. Neurocognitive endophenotypes (endophenocognitypes) from studies of relatives of bipolar disorder subjects: a systematic review. Neurosci Biobehav Rev. (2008) 32:1426–38. 10.1016/j.neubiorev.2008.05.019 18582942

[16] Doyle AE Wozniak J Wilens TE Henin A Seidman LJ Petty C. Neurocognitive impairment in unaffected siblings of youth with bipolar disorder. Psychol Med. (2009) 39:1253–63. 10.1017/S0033291708004832 19079809 PMC2853769

[17] Kéri S Kelemen O Benedek G Janka Z. Different trait markers for schizophrenia and bipolar disorder: a neurocognitive approach. Psychol Med. (2001) 31:915–22. 10.1017/S0033291701004068 11459389

[18] Kulkarni S Jain S Janardhan Reddy YC Kumar KJ Kandavel T. Impairment of verbal learning and memory and executive function in unaffected siblings of probands with bipolar disorder. Bipolar Disord. (2010) 12:647–56. 10.1111/j.1399-5618.2010.00857.x 20868463

[19] Nehra R Chakrabarti S Pradhan BK Khehra N. Comparison of cognitive functions between first- and multi-episode bipolar affective disorders. J Affect Disord. (2006) 93:185–92. 10.1016/j.jad.2006.03.013 16678909

[20] Trivedi JK Goel D Dhyani M Sharma S Singh AP Sinha PK. Neurocognition in first-degree healthy relatives (siblings) of bipolar affective disorder patients. Psychiatry Clin Neurosci. (2008) 62:190–6. 10.1111/j.1440-1819.2008.01754.x 18412842

[21] Alda M Kapczinski F. Staging model raises fundamental questions about the nature of bipolar disorder. J Psychiatry Neurosci. (2016) 41:291–3. 10.1503/jpn.160151 27575857 PMC5008917

[22] Tatay-Manteiga A Balanzá-Martínez V Bristot G Tabarés-Seisdedos R Kapczinski F Cauli O. Clinical staging and serum cytokines in bipolar patients during euthymia. Prog Neuropsychopharmacol Biol Psychiatry (2017) 77:194–201. 10.1016/j.pnpbp.2017.04.028 28445689

[23] Balanzá-Martínez V Tabarés-Seisdedos R Selva-Vera G Martínez-Arán A Torrent C Salazar-Fraile J. Persistent cognitive dysfunctions in bipolar - I and schizophrenic patients: a 3-year follow-up study. Psychother Psychosom. (2005) 74:113–9. 10.1159/000083170 15741761

[24] Correa-Ghisays P Balanzá-Martínez V Selva-Vera G Vila-Francés J Soria-Olivas E Vivas-Lalinde J. Manual motor speed dysfunction as a neurocognitive endophenotype in euthymic bipolar disorder patients and their healthy relatives. evidence from a 5-year follow-up study. J Affect Disord. (2017) 215:156–62. 10.1016/j.jad.2017.03.041 28334676

[25] Rosa AR Sánchez-Moreno J Martínez-Aran A Salamero M Torrent C Reinares M. Validity and reliability of the functioning assessment short test (FAST) in bipolar disorder. Clin Pract Epidemiol Ment Health (2007) 3:5. 10.1186/1745-0179-3-5 17555558 PMC1904447

[26] Rosa AR Magalhães PV Czepielewski L Sulzbach MV Goi PD Vieta E. Clinical staging in bipolar disorder: focus on cognition and functioning. J Clin Psychiatry (2014) 75:450–6. 10.4088/JCP.13m08625 24922497

[27] Sánchez-Morla EM Barabash A Martínez-Vizcaíno V Tabarés-Seisdedos R Balanzá-Martínez V Cabranes-Díaz JA. Comparative study of neurocognitive function in euthymic bipolar patients and stabilized schizophrenic patients. Psychiatry Res. (2009) 169:220–8. 10.1016/j.psychres.2008.06.032 19758705

[28] Lewandowski KE Cohen BM Ongur D. Evolution of neuropsychological dysfunction during the course of schizophrenia and bipolar disorder. Psychol Med. (2011) 41:225–41. 10.1017/S0033291710001042 20836900

[29] Post RM Fleming J Kapczinski F. Neurobiological correlates of illness progression in the recurrent affective disorders. J Psychiatr Res. (2012) 46:561–73. 10.1016/j.jpsychires.2012.02.004 22444599

[30] Czepielewski LS Massuda R Goi P Sulzbach-Vianna M Reckziegel R Costanzi M. Verbal episodic memory along the course of schizophrenia and bipolar disorder: a new perspective. Eur Neuropsychopharmacol. (2015) 25:169–75. 10.1016/j.euroneuro.2014.09.006 25311898

[31] Fernandes BS Berk M. Staging in bipolar disorder: one step closer to precision psychiatry. Rev Bras Psiquiatry (2017) 39:88–9. 10.1590/1516-4446-2017-3902 28591270 PMC7111440

[32] Kessing LV Andersen PK. Evidence for clinical progression in unipolar and bipolar disorders. Acta Psych Scand. (2017) 135:51–64. 10.1111/acps.12667 27858964

[33] Passos IC Mwangi B Vieta E Berk M Kapczinski F. Areas of controversy in neuroprogression in bipolar disorder. Acta Psychiatr Scand. (2016) 134:91–103. 10.1111/acps.12581 27097559

[34] López-Jaramillo C Lopera-Vásquez J Gallo A Ospina-Duque J Bell V Torrent C. Effects of recurrence on the cognitive performance of patients with bipolar I disorder: implications for relapse prevention and treatment adherence. Bipolar Disord. (2010) 12:557–67. 10.1111/j.1399-5618.2010.00835.x 20712758

[35] Robinson LJ Ferrier IN. Evolution of cognitive impairment in bipolar disorder: a systematic review of cross-sectional evidence. Bipolar Disord. (2006) 8:103–16. 10.1111/j.1399-5618.2006.00277.x 16542180

[36] Lee RS Hermens DF Scott J Redoblado-Hodge MA Naismith SL Lagopoulos J. A meta-analysis of neuropsychological functioning in first-episode bipolar disorders. J Psychiatr Res. (2014) 57:1–11. 10.1016/j.jpsychires.2014.06.019 25016347

[37] Samamé C Martino DJ Strejilevich SA. Longitudinal course of cognitive deficits in bipolar disorder: a meta-analytic study. J Affect Disord. (2014) 164:130–8. 10.1016/j.jad.2014.04.028 24856566

[38] Strejilevich SA Martino DJ. Cognitive function in adulthood and elderly euthymic bipolar patients: a comparison to test models of cognitive evolution. J Affect Disord. (2013) 150:1188–91. 10.1016/j.jad.2013.05.012 23726659

[39] Bora E. Developmental trajectory of cognitive impairment in bipolar disorder: comparison with schizophrenia. Eur Neuropsychopharmacol. (2015) 25:158–68. 10.1016/j.euroneuro.2014.09.007 25261263

[40] Bora E Hidiroglu C Özerdem A Kaçar ÖF Sarisoy G Civil Arslan F. Executive dysfunction and cognitive subgroups in a large sample of euthymic patients with bipolar disorder. Eur Neuropsychopharmacol. (2016) 26:1338–47. 10.1016/j.euroneuro.2016.04.002 27139077

[41] Jensen JH Knorr U Vinberg M Kessing LV Miskowiak KW. Discrete neurocognitive subgroups in fully or partially remitted bipolar disorder: Associations with functional abilities. J Affect Disord. (2016) 205:378–86. 10.1016/j.jad.2016.08.018 27573491

[42] Librenza-Garcia D Kotzian BJ Yang J Mwangi B Cao B Pereira Lima LN. The impact of machine learning techniques in the study of bipolar disorder: a systematic review. NeurosciBiobehav Rev. (2017) 80:538–54. 10.1016/j.neubiorev.2017.07.004 28728937

[43] Bauer IE Wu MJ Frazier TW Mwangi B Spiker D Zunta-Soares GB. Neurocognitive functioning in individuals with bipolar disorder and their healthy siblings: a preliminary study. J Affect Disord. (2016) 201:51–6. 10.1016/j.jad.2016.04.026 27179338 PMC4899217

[44] Doucet GE Bassett DS Yao N Glahn DC Frangou S. The role of intrinsic brain functional connectivity in vulnerability and resilience to bipolar disorder. Am J Psychiatry (2017) 174:1214–22. 10.1176/appi.ajp.2017.17010095 28817956 PMC5711589

[45] Ratheesh A Lin A Nelson B Wood SJ Brewer W Betts J. Neurocognitive functioning in the prodrome of mania-an exploratory study. J Affect Disord. (2013) 147:441–5. 10.1016/j.jad.2012.09.017 23141631

[46] Fries G Vasconcelos-Moreno M Gubert C dos Santos B Sartori J Eisele B Hypothalamic-pituitary-adrenal axis dysfunction and illness progression in bipolar disorder. Int J Neuropsychopharmacol. (2014) 8:pyu043 10.1093/ijnp/pyu043 PMC4368875 25522387

[47] Pfaffenseller B Wollenhaupt-Aguiar B Fries GR Colpo GD Burque RK Bristot G. Impaired endoplasmic reticulum stress response in bipolar disorder: cellular evidence of illness progression. Int J Neuropsychopharmacol. (2014) 17:1453–63. 10.1017/S1461145714000443 24800824

[48] Andreazza AC Kapczinski F Kauer-Sant'Anna M Walz JC Bond DJ Goncalves CA. 3-Nitrotyrosine and glutathione antioxidant system in patients in the early and late stages of bipolar disorder. J Psychiatry Neurosci. (2009) 34:263–71. 19568477 PMC2702443

[49] Kauer-Sant'Anna M Kapczinski F Andreazza AC Bond DJ Lam RW Young LT Brain-derived neurotrophic factor and inflammatory markers in patients with early- versus late-stage bipolar disorder. Int J Neuropsychopharmacol. (2009) 12:447–58. 10.1017/S1461145708009310 18771602

[50] Balanzá-Martínez V Crespo-Facorro B González-Pinto A Vieta E. Bipolar disorder comorbid with alcohol use disorder: focus on neurocognitive correlates. Front Physiol. (2015) 6:108. 10.3389/fphys.2015.00108 25904869 PMC4387475

[51] Dias VV Balanzá-Martinez V Soeiro-de-Souza MG Moreno RA Figueira ML Machado-Vieira R. Pharmacological approaches in bipolar disorders and the impact on cognition: a critical overview. Acta Psychiatry Scand. (2012) 126:315–31. 10.1111/j.1600-0447.2012.01910.x 22881296

